# Southern Dental Association

**Published:** 1886-11

**Authors:** 


ARTICLE II.

### SOUTHERN DENTAL ASSOCIATION.

EIGHTEENTH ANNUAL MEETING.

AT NASHVILLE, TENN., JULY 27, 28, 29 AND 30.

(Reported by Mrs. M. W. J.)

[Continued from October Number.]

Prof. Taft was called for, and greeted with applause.

He said that he had no extended remarks to make, though all were interested, and all had an opinion on this subject; a man was no longer excusable who had not some opinion, though of course they varied widely. As to the pathology—but first of all nomenclature; names are adopted and become fashionable or customary, which are not appropriate.

There are different manifestations in different cases of this disease. The name Riggs' disease is not appropriate; he had no idea of pre-empting it; he made no special claims to discoveries; his system was only an extension of what had already been done. The name Pyorrhœa Alveolaris is stilted, and has no special meaning beyond that of a flow of pus from the alveolus; the old term "conjoined suppuration of gums and alveolus" is perhaps better. It is not a specific disease; it has many phases—the peculiar separation of the gums from the teeth; the flow of pus; the formation of pockets; the channel, down the roots of the teeth, sometimes penetrating very deeply, and sometimes only superficial, and sometimes extending down to the apex. A good deal has been said as to the disease being local or constitutional. There is undoubtedly a systemic condition which favors its occurrence and continuance, but yet it is undecided what that condition is. Whoever says *always* should study this up. There may be a poison in the system which favors this disease under local stimulus, though not without local irritants. "Conjoined suppurations of the gums and alveolus" does not exist solely and alone from systemic conditions, independent of local causes; the two act together. Some agents expend their great force on the periosteum as mercury in ptyalism, without local irritant beyond itiated saliva. Riggs said there was *always* a local irritant. There was wonderful success in his treatment, which rarely ever failed, though he seldom employed systemic treatment. Usually, in all cases there is more or less of local irritation; it may be vitiated debris of food or epithelial scales, or salivary calculus or serumal calculus, or the margin of the alveolus may be diseased in some form disintegrating as in necrosis of the margin. Then microbes, here as elsewhere, play an active part; what the results are of the presence of these organisms is not yet clearly defined; some believe they prey on the living tissue and break it down; others that their fermenting products work the mischief. There is room here for study and investigation; nobody has yet brought out a tangible point on which we can rest satisfied. I do not believe they are always there, but though they are present in many cases, in some of the most severe cases, no vestiges of organisms are found in the debris. The use of a germicide is, however, entirely admissable, and should be resorted to. When we know what we are dealing with, we know what is needed—(Time up.) (Go on, go on.) Different phases or stages of the affection require different modes of treatment, and it is important to differentiate. Some features are common to all cases. The removal of all irritants has been spoken of. The first essential is the local purification of the mouth and teeth, which must then be kept in a clean condition; all calcareous deposits must be removed, the pockets being probed deeply with explorers; all sloughing and decayed portions of gum tissue, and all necrosed portions of the alveolus must be removed with appropriate instruments. A large share of the instruments sold for this purpose are entirely unfit; they should be made slender, and should chisel downwards, removing every particle of debris; this will not be found difficult with proper instruments, but cannot be done with a great hoe or by digging down. All necrosed, roughened portions must be removed; with the engine all ragged points can be burred off. The use of sulphuric acid has been referred to; this only acts upon tissues that are in a dying condition; healthy tissue is safe. Granulation cannot attach where there is the least deposit. We hear of mixing this, that, and the other, but you must know what you are aiming at, and why you should use this remedy rather than that, and not combine two, one of which will undo the work of the other. When a mechanic selects a tool he knows what he wants it to do, and selects the one adapted to do that one thing. There is too much ignorant use of therapeutic agents; very little medication is needed in any case. Riggs used no medicine at all, in many cases; sometimes a little tannin or carbolic acid, but he did not use heroic treatment. Iodine in creosote Atkinson called his *big nigger*; he now uses a mild solution of salycilic acid. As a rule we should rely more on surgical than on therapeutic treatment; study causes and systemic peculiarities, but rely on the thorough removal of debris. We have sloughing, half-dead tissue, instead of healthy socket; even the surface of the gums require scraping off, the exudations being a continual irritant. Peroxide of hydrogen is the *grand* agent, though I don't say "don't use anything but that." It prevents decomposition of debris; it is a gerimicide for organisms which are destroyed and thrown out There will be *healing* after thorough surgical treatment, though I don't say there will be restoration of tissues or process. If the teeth are loose the gums cannot be healthy; pabulum is thrown out, but the granulation is continually broken up and disturbed; the teeth must be bound with fine suture wire and made firm and secure; this alone will soon relieve irritation. In a word, remove *all* irritants, and dress with a little peroxide of hydrogen, or salycilic acid in alcohol.

Dr. Thackston said that with hearty sincerity he wish ed to thank his friend Taft for his able address. There is one point to make; when in the domain of speculative theory it is easy to map out forces, causes and results, but in the domain of facts, what we have most carefully thought out sometimes fails us. Our friend, in his clear and happy manner, placed great stress on the removal of local irritants; but where do these irritants, this local debris, come from? We have all seen conjoined suppuration of the gums and alveolar process occur where we know the most scrupulous care has been taken of the mouth and teeth. I have seen a professional brother whose reputation is well established for great manipulative skill, employ in vain every resource for the arrest of this disease; there is an acrid, dissolving exudation, which we cannot arrest, and before which all goes; the gums and alveolus melt away and the teeth are lost. Salivary calculus has its source of origin in the secretions of the mouth, and that can be kept off, but there is another source of deposits which we have not reached. Uric acid is a happy suggestion which I am glad to hear made. Teeth which are perfectly free from caries loosen and drop out, having resisted all remedial agents. The neck and sides of the root may be clean, but the very apex be found enveloped in a reddish brown deposit of a tartarous variety; not from the saliva, not from the gums—but from the blood itself; we can't get at it, and the tooth is lost.

Dr. Winkler: Do you infer that the deposit at the apex takes place previous to the loosening of the gums? that it begins at the apex?

Dr. Thackston: I do, sir; there are two sources of calculus; this form is not a salivary calculus; the mouth is not the only place where calculi are deposited; we find them in the liver and elsewhere. The reddish brown incrustation at the apex ot the roots is not from the gums, it is from the blood; and is systemic, and would eventually cause the loss of all the tveth.

Dr. Teague: Would it not be possible to reach this deposit at the apex with instruments? Could they not be run down to the deposits?

Dr. Thackston: No, sir; teeth may have this deposit at the apex, which are firm in the socket, and the gums well attached at the necks.

Dr. W. C. Wardlaw, (calling Dr. Catching to the chair): I have seen cases with this dark, sanguinary deposit on the roots. I gave one to Dr. Frank Abbott to examine with the microscope; he had several specimens. This occurs without any detachment of the gums.

Dr. Taft: One word in explanation. There are extreme cases, I have seen cases similar, but never in the absence of alveolar abscess. The gums may be firmly attached at the neck, and serumal calculus be found upon the root, but not in one single instance have I found it, unless there had been alveolar abscess. The exception must be very rare, and we need to deal with every day cases, not with the exceptions. There are some cases which cannot be explained, but they are rare.

Dr. Rembert, (Natchez,): Is not serumal calculus thrown out from degenerated plasm, or pus flow, as salivary calculus is precipitated from saliva? Serumal calculus is not from the blood, but from serum a product of decomposition exuding from the blood through the tissue, and from the blood only in that sense; a product or deposit from decomposing serum.

Dr. J. J. R. Patrick said that chemical analysis had never shown any difference between sanguinary and salivary calculus; it is only the mode of deposit, or of throwing out, that is different. That from the saliva is secreted and thrown out; the blood vessels are unhealthy and the capillaries ruptured in throwing out serum. Salivary calculus is a secretion; sanguinary calculus is a concretion.

The time for adjournment having arrived, Dr. Catching suggested that a night session be held, as there were other papers to be read.

Dr. Salomon said that arrangements had been made for Dr. J. R. Knapp's night clinics with electric light and apparatus.

Dr. J. Hall Moore understood that it had been agreed to by the committee of Arrangements, that the members visit the museum of the Tennessee Historical Society at 8 P. M.

After some discussion it was decided to have a night session.

Adjourned to 8 P. M.

---

THURSDAY, July 29.

Called to order at 8 P. M., Dr. B. H. Catching in the chair.

Dr. W. R. Bourne, Hopkinsville, Ky., was elected to membership.

The discussion of Pathology and Therapeutics continued:

Dr. Morgan said that the fundamental principle in the treatment of disease is the removal of the cause. Every step taken should be based on the idea of its pathology differentiated in the mind of the practitioner. The only radical cure for this disease (Pyorrhœa Alveolaris) is the removal of the teeth. He had never known it to fail; and this suggests the question whether the cause does not lie in the teeth themselves; whether the primary cause is located in the tooth or its environments? Some writers say the tooth is composed of enamel, dentine and cementum; others include the pulps; others again include the investing membrane of the tooth. The trouble seems to be located either in the cementum or in the pericementum, or perhaps back of that, as it is hereditary to a large extent. One reason why it is probably located in the cementum, is that if the cementum is thoroughly scraped off, and thus devitalized by separation from the periosteum, passing low down on the roots of the teeth till the point of juncture is reached, the trouble is removed, and the gums heal up around the tooth. All irritating substances tend to produce absorption. Nature takes that method of relieving herself of trouble from pressure. Whatever may be the etiology pathologically we have:

First—irritation,
Then—inflammation,
Then—solution of parts.

The incrustations are not always calcareous; soft, pasty matter, causes breaking up of tissues, followed by a darkish tartar, the result of decomposition of the tissues of the blood. Lime salts are carried in the blood, and in this breaking up, the lime salts, which have to be disposed of, crystalize about the neck of the tooth, crystalizing under the laws of chemistry.

Almost everv one who has spoken mentioned removing necrosed alveolar process. In treating the disease for nearly forty years, I have not seen exceeding six cases where there was any necrosed bone. There is softening of the bone, loose lime salts are carried off by the emunctories —perhaps used in building up other portions of the body, though we don't know that. We find softened alveolar process, but not necrosis. The process is all absorbed, which is a physiological action. Necrosis is pathological, and cannot undergo a physiological process. Physiological processes relate to living tissues; dead tissues are not absorbed. In this disease the alveolus is dissolved—brought into solution and carried off in the general circulation. In a case recently seen, a man of bilious temperament, sinewy, tough and elastic, the left inferior lateral incisor was loose in its socket; removing all debris the exposed edges of the alveolus were reached and found to be necrosed, and only this one tooth so affected.

One of the grand mistakes in the treatment of this disease occurs in the too frequent surgical operations. A tooth gives evidence of pyorrhœa; there are foreign substances around the root. These are removed as clean as possible, medicaments applied, and the patient told to come again to-morrow for a renewal of the process. If the work has been thoroughly done at the first sitting, the soft tissues are broken up, and blood is poured out; a fibrous coagulum is formed which should be retained there; it furnishes protoplasm, and should not be disturbed. It is similar to what takes place when a tooth is extracted; blood is coagulated, the color is washed out, the coagulum grows tough and a fibrous tissue is produced. About the third day the socket will be found free from blood and filled with organized lymph traversed with little blood vessels, which soon becomes perfectly organized soft tissues. In the same way, if the pockets are left alone, say for ten days, nature will fill them up; gentle friction upon the gums is advisable. If here and there we find some trouble, it is proof that the debris was not thoroughly removed—but only those points should be touched a second time. The question was asked. Is the periosteum ever produced? is the cementum ever re-produced? *Never*, NEVER. Nature does not do that. The tissue that is formed is not normal tissue; it is largely of the nature of scar tissue; it is of lower vitality takes on inflammation more slowly, and breaks down more slowly. Nature builds up a scar tissue, analogous to cartilage, which holds the tooth firmly in place. The cavity made by an abscess is filled up by comparatively healthy tissue; but it is never perfect; there is always a little chronic inflammation at the end of the root—conditions which normal tissue would not tolerate. If fungus cells are found in the walls of the pockets, nothing equals carbolic acid for destroying them by escharotic action, also stimulating healthy granulation. Robinson's remedy, carbolic acid and caustic potash, is also very effective. When two or three teeth have been thoroughly cleaned of debris, by Riggs' method, a roll of cotton or fibres of candlewick, is dipped in the solution and laid on paper to drain. It is then placed around the necks of the teeth, and left there while two or three more teeth are cleaned. When ready to apply the remedy to these, remove the cotton from the first. It gives a charred appearance, destroys all fungus growth and pus. These teeth should not be touched any more, except brushing with a very soft brush. If not healthy after this treatment, it is because they were not thoroughly cleaned. But in any case, wait eight or ten days before renewing the application. The disease seems to be local, but it is the local expression of systemic disease. Whole families, parents and children alike, are affected by it. If in the case of young children, it is taken in hand promptly, say by eight years of age, it may sometimes be arrested forever, with strict cleanliness and friction of gums. It is much more prevalent than might be supposed, though not perhaps as much here as has been described in Georgia. The constitution is undermined and destroyed by it. Physicians don't understand it. The vital forces are reduced, and patients succumb readily to other diseases, through lowered vitality. In a recent case, an old man of seventy was supposed to be dying of hereditary consumption. He was thin and bloodless, and scarcely able to walk across the yard. He had nearly all his teeth which were badly affected by pyorrhœa. Within three months after their extraction, his cough and expectoration were much lessened, his night fevers left him, and he gained ten pounds in flesh. In another case a lady gained forty pounds in weight after the teeth were removed. Nothing was done for a cure beyond taking out the teeth. The disease is akin to scurvy, and diet must be carefully looked to. Feed on the fat of the land, so that it is digestible.

Dr. J. Hall Moore asked if the cases cited could not have been treated and cured without the removal of the teeth?

Dr. Morgan replied that there was always a predisposition to return; the hereditary tendency cannot be removed; it is the local manifestation of constitutional disease.

Dr. Rembert, Natchez, asked if the calcareous deposits in young subjects, were equal in proportion to older subjects?

Dr. Morgan replied that it differed both in quality and quantity. The deposits in young patients are not dense or calcific; they are of a granulated, cheesy consistence—only semi solid. Cotton loaded with carbolic acid, or iodine or other mild escharotie, laid under the loose gums, and left there till expelled by nature, will often check the ichorous discharge and relieve it entirely. Tonic treatment is also needed, but any systemic treatment with a view to the general health, should be turned over to the general physician. It is not the province of the dentist to treat disease.

Dr. Rembert thought that even heredity might be overcome.

Dr. Morgan: Only feeble conditions and forms of disease were overcome—not the hereditary tendency to recurrence.

Dr. J. Hall Moore wished to call attention to a point overlooked by previous speakers. He had not seen a single case which had made much progress, that was not complicated with catarrh, and in that case, constitutional disease could not be cured by mere local treatment. In many cases there were no deposits on the teeth; the roots were perfectly clean, bright and polished, but all other symptoms—exuding pus, pockets reaching down to the apex, roughened alveolar border, etc., but neither salivary nor sanguinary tartar, and always complicated with catarrh.

He knew of one case which had been published in every journal in the country as evidence of the success of Dr. Riggs' method of treatment, (in the hands of Dr. Mills) but the patient eventually lost every tooth, as clean as a pitcher, and perfectly sound; with the exception of three or four small cavities the teeth were sound and hard. This patient had catarrh fearfully. Such cases cannot be cured by local treatment, which is at best, only palliative. Dr. Moore requested the members of the Association to note the association of catarrh and pyorrhœa in their practice. He was apprehensive that there was no radical cure for the disease; that it would always break out again, unless catarrh was first cured, the latter being the cause of the trouble. He had submitted the idea to Dr. Riggs who had agreed with him.

Dr. Rembert asked whether it were not a mooted question that catarrh is constitutional?

Dr. Moore said it was not, in his mind at least.

Dr. Morgan said that Pyorrhœa Alveolaris obtains much more largely in the scrofulous diathesis, whether in the blood or in the lungs, and was also more frequent in blondes. He would not attempt the cure of such cases until a physician had given constitutional treatment.

On motion of Dr. Beech the subject was passed.

Dr. Beech stated that Dr. Knapp was now ready for his clinics with the electric lamps, and moved that the Association adjourn, to witness the clinics.

The President stated that there would be time for the reading of a short paper, while Dr. Knapp was arranging his battery, etc.

Dr. B. L. Byrnes, Memphis, said that in reading the DENTAL JOURNAL, he had always found the chapters of Office Incidents very interesting. He had accordingly made that the subject of his paper, which was then read.

9:30 P. M.—The clinic of Dr. J. Rollo Knapp was quite largely attended, considering the hour. The electric appliances consisted of a number of lamps varying in power from 27 candle to ¼ candle. The larger sizes from three candle up were designed for use out of the mouth. By an ingenious invention the lamps were held upon the forehead, and the light for night work directed with great precision, the lamps being ignited or extinguished instantly by means of a thumb screw situated near the right ear. Each lamp was provided with a reflector, which entirely screened the eyes of the operator, at the same time throwing the rays of light directly where they were most needed. The smaller lamps were so constructed as to permit of use in the mouth, a lamp being attached either to the tooth to be operated upon, or to a contiguous tooth, as convenience may suggest. The light may be used in the mouth for an indefinite length of time without heating or burning the mouth. There were also other electric appliances of original and varied character, which called forth the highest encomiums from all who saw them.

Adjourned to 9 A. M.

---

FRIDAY, July 30.

FOURTH DAY—MORNING SESSION.

Called to order at 9 A. M., the President in the chair. Dr. O. Salomon, acting Secretary.

Owing to the absence of the Recording Secretary, roll-call and the reading of the minutes were dispensed with.

Dr. W. H. Morgan stated that he had something of a report to make, the items of which were in the hands of

Dr. G. F. S. Wright, of the Executive Committee, who was not present.

If there was no other business before the Association, he would make a verbal report of a matter, which though his business, was not his pleasure.

Charges had been preferred of unprofessional and ungentlemanly conduct on the part of one of the members of the Association, in the form of Resolutions of Impeachment. It was the right and duty of the President to appoint a committee to investigate these charges; and report at the next annual meeting.

The President: The gentlemen have heard the remarks of Dr. Morgan. If there is no objection I will appoint as the committee of investigation:

Drs. W. W. H. Thackston, J. Hall Moore, G. H. Winkler.

Resolutions of thanks were then offered to the Y. M. C. A., Vanderbilt University; the Tennessee Historical Society; the Nashville Art Association, and to Mr. Carroll, in charge of the latter, for his kind attentions to the Association. Carried by unanimous rising vote.

Drs. Salomon, Morgan and Crenshaw, the committee appointed to draft resolutions on the deaths of Drs. Holmes, Best, Jobson and Redman, reported as follows:

(No report was furnished.)

The discussion of Pathology and Therapeutics continued:

Dr. ———— said that though not a member of the profession, he had been an interested listener, and had never enjoyed a meeting more. He thought it would be of great benefit to members of the medical profession to attend these meetings. The discussion of diseases of the gums and alveolus had been very valuable. His own experience in that respect had been somewhat peculiar. Having had his teeth examined sometime before, they had been found in perfect order with the exception of a little calculus, and stains from the use of tobacco. He then concluded to abandon the use of tobacco, but in a very short time his teeth got loose, with pus exuding from the gums, etc. He resumed the use of tobacco, and his teeth got perfectly firm again and the gums sound and healthy. He wished to know what connection, if any, there was between the use and disuse of tobacco, and the condition of his teeth? He had had no treatment of any kind.

No further remarks were made, and on motion the subject was passed.

### CHEMISTRY.

Dr. Theo: Johnstone, South Carolina, read a paper entitled

### GOLD—COHESIVE AND NON-COHESIVE.

There being no other papers, the subject was declared open for discussion.

The Executive Committee recommended for membership Dr. R. Paul Jones, ————, who was duly elected.

Dr. Morgan said South Carolina ought to cultivate the author of such a paper, and he congratulated the Association on having a young member capable of producing it. He must, however, differ with him in some points. It is not generally received that gold is the most ductile of metals, but that is immaterial. The next misstatement is that gold must be chemically pure in order to weld perfectly. Gold with 6 per cent. silver alloy is very cohesive, as for instance, that made by Abbey Bros. The color indicates the presence of silver. But the purer it is the more cohesive it is. We want it pure, and he hopes we will get it pure. The writer of the paper assumed that latent heat was driven off by the hammer. The heat is produced by the hammering; it is the result of friction, but heat is not actually driven off. There is as much heat present in solid gold as when the particles are loose, but it is not manifest. When a bar of solid gold is hammered out, heat is developed as the result of friction among the molecules.

Dr. Marshall, Little Rock, asked what per centage of platinum gold would receive without impairing its cohesive properties.

Dr. Morgan replied that he had not experimented in that direction. Had never used it when welding was necessary; only for artificial dentines. One pennyweight to the oz. would stiffen it and make it harder; two pennyweights to the oz. gave a spring temper equal to steel if not annealed.

Dr. Johnstone said that there was a great deal of speculation about heat; we can't see it; we can only see its effects. In all matter the pores are occupied by heat. If a sponge is dipped in water the pores are at once filled with water, which can readily be squeezed from it. In the same way heat occupies the pores of gold, causing its expansion, when hammered the heat is driven out, as when the sponge is squeezed. In iron this can be more readily seen, the metal turning red under the hammer.

Dr. Salomon: In reply to the question put by Dr. Marshall—stated that the addition of platinum to gold in the proportions of three, six or even ten per cent. did not destroy the welding properties. Ten per cent. makes it very hard, but three or six per cent. is easily worked, and very cohesive.

Dr. Winkler said that platinum and gold cylinders, where the platinum is beaten out and covered with gold which is sweated to it, are very cohesive, and easily welded. They are used where gold alone would not resist the wear. No. 3 cylinders are liable to break instruments, but the work has a beautiful appearance, making front fillings which are scarcely perceptible to the eye, though the third of a tooth is built down with it.

Dr. Morgan said the heat evolved was simply in obedience to the law of equilibrium; but the heat was not an entity as held by the old philosophy; it does not occupy space, it is only a mode of motion, it is eliminated or produced by the motion of the molecules. If gold and platinum are melted together, the molecules lose their cohesion, and the molecules of one metal being smaller than those of the other, they run in and pass between each other. If molten gold is stirred with a platinum wire, the platinum wastes away; its molecules floats off and pass between the molecules of gold.

On motion CHEMISTRY was passed, and

OPERATIVE DENTISTRY, which had been temporarily passed, was called up again, at the request of Dr. Crenshaw, who said that he had had no opportunity of answering the remarks made on the discussion of his paper. He said that though, as Dr. McKellops claimed, some men had abandoned the electric mallet, it was not true of the most prominent men. The electric mallet is a complicated affair, the battery must always be exactly right; the use of the mallet and the care of its different parts is hard to understand; too much for ordinary men, and few are willing to undertake the care and annoyance necessary to keep it always in order. He has had tussles with it himself, with both the mallet and the battery, but when kept in proper order, and the gold all right, it is the finest device ever invented. The fault does not lie in the mallet, but in the operator, who has not the ability to keep it in order. He had brought his own mallet,, superbly adjusted, for a clinic, but having a new battery it wouldn't go. To be always ready, it is necessary to have two, one at the manufacturers, and one to use, alternately. A new mallet costs $35.00, but he had bought one for $10.00, and with $17.00 out for repairs it was as good as new. The blow of the electric mallet is philosophically correct; the blow of the mechanical mallet is not correct; you can drive a tack down with it. You can with the pneumatic mallet, and you can with the electric mallet, with proper sweep. Again, Dr. McKellops said that he had seen the whole socket removed by separators. He probably meant some of the bone, for I don't see how the socket could be removed,* though

* The word used by Dr. McKellops was *ruined*, not *removed*—Reporter.

I have seen the suture opened by separating teeth in children. We must use judgement and sense, or we may do harm. A patient for whom I made separations yesterday was well pleased, and found it less painful than rubber wedges. Dr. Winkler said that soft gold was more acceptable to cervical margins. I have run the gauntlet of all the different methods, hand and automatic pressure, matrices, soft gold, hard gold, and both combined, but don't like the idea of changing from one material to another in the same cavity; you don't get proper welding of the two. Why use soft foil? You admit that by weight you can put in more cohesive gold than soft; this indicates that a filling of cohesive gold is more dense. Why does Dr. Winkler put soft foil at the margins? It is done because it is so difficult to get at the margins with cohesive gold. But if the tooth is cut down, and access obtained, and the dam applied, cohesive gold will give better results. I respect those men but I antagonize their views.

Dr. Morgan: You say we all know what the blue appearance around the filling means. We *don't* always know.

Dr. Crenshaw: It always means a recurrence of decay.

Dr. Morgan: Exactly; *if* it has not been properly filled.

Dr. Crenshaw: If the filling is made with chunks and pellets of foil, and not properly condensed, of course the work is faulty. I do not hope to make converts, or to convince any one, but merely to lay before you what I *believe*; not to present *new* principles, but the principles which have been adopted by such men as Atkinson, Marshall Webb and Parmly Brown. I hoped to elicit a discussion, but I did not expect to convince any one. I know I havn't done it, but I don't care a bit.

Dr.—Beech thought it was a narrow view to hold that every cavity, under every condition, should always be filled in the same way. He thought that there were many teeth that could not be filled with cohesive gold, that might be saved with something else.

Dr. Crenshaw: If an angle has to be turned, it cannot be done with soft gold; the filling cannot be made to stand without a wall. Everything that can be done with anything else can be done as well, if not better, with cohesive gold and the electric mallet, and much more that cannot be done with soft foil—Dr. Webb never used soft foil; Dr. Brown never uses soft foil; but it is not because these eminent men don't know how; but some men cannot do it, they don't know how, they never will be able. If it is possible to make a system, it can only be by confining to cohesive gold, to simplify and avoid confusion. Dr. Winkler was looking for a subject for his clinic. A gentleman offered an approximal cavity in a bicuspid, with angle turning and going into the crown; oh, no! he wouldn't take that—he wanted something he could fill with soft foil.

I have used soft foil, myself, with fair results, but I can do much better with cohesive gold and mallet force.

Dr. Winkler: Dr. Crenshaw spoke of chunks and billets, etc. We very often see fillings looking as if the gold had been chunked right in, but some men run after new gods, and are so carried away by the latest novelties that sense cannot be knocked into their heads with either billets or chunks! We use soft foil at the cervical walls, *not* because it is more acceptable, but because with it we are enabled to preserve poor, soft teeth from decay. It is true that the use of cohesive gold requires extreme manipulative skill, and that ordinary operators will have better results in saving teeth, if they use soft gold at the cervical walls. It saves much painful labor to the operator, and much suffering to the patient. It is true that Marshall Webb said that tin foil was the most difficult material to use, but though I revere and respect him as a great man, it must be that he deemed himself not able to use soft foil, or he would not have put in his book that tin foil was the most difficult.

As to my clinic, I was invited to clinic with soft foil. The case mentioned by Dr. Crenshaw was one which, to give the best results, required the combination of both cohesive and soft gold, and I was not there for that purpose. I did not want to use cohesive gold, when I was up for a clinic for soft gold. The operation would have been simple and pretty, filling two-thirds full with soft foil, which could have been completed in three or four minutes, and then built out with cohesive gold, which welds perfectly, as has been proved and demonstrated, by heating small pieces and putting on while hot. The soft foil must be thoroughly condensed, shaping it to conform to the surface of the tooth, preserving the relations of the cavity walls. It is the duty of every gentleman to study up the different methods. Dr. Crenshaw said he had tried everything, but when the inevitable blue line appeared, it showed he did not know how to use soft foil.

Dr. McKellops: As far as the electric mallet is concerned, I have got something to say. The gentleman presumes to say that Perry, Darby, Allen and myself can't take care of a mallet! that we don't know our business! I have had as good a mallet as was ever made, and the best batteries. I have a girl who takes splendid care of everything, and everything I have is always perfectly clean and in perfect order. My objection to the electric mallet is that it is too harsh, too severe; patients can't sit under it. Of course, it is a great nuisance, but I have proper assistants to care for all that. Perry has the very best of everything. I did not say anything about the use of Perry's separators; I said by rapid wedging, but I have seen Perry himself use wedges instead of his own separators. Separating with tape creates very little soreness, but forcing apart with separators and screws is very painful. The socket I spoke of was ruined by driving a stick in.

Don't say I can't take care of my apparatus. I like Bonwill's instruments, but I don't like the man who condemns me because I won't give him a certificate. The beauty of Bonwill's instrument is that the blow is perfect. You will be converted in a minute, if you try his mallet, but you have got to learn how to use it. I didn't like it myself at first. I changed the springs myself, so as to use it with either hand. The electric mallet delivers 2,000 blows, but Bonwill's will make 3,000 in a minute. I did not abandon the electric mallet because it was a nuisance, though it has to be in Philadelphia all the time unless you have proper assistants. I have recently seen a new one, which is very small and light, but you must have a battery.

I like the platinum foil. I first learned of it from the *Scientific American*, and sent to Black, of California, and got his, which is very heavy. I use it for all work which shows, and which must stand mastication; for powerful jaws which grind off the surface, platinum gold is like steel. You can also match the color of the tooth very exactly. I have recently built down all the front teeth for a very large man, who is very blonde. He shows his teeth very much, but you can't tell that they are filled. The color never changes, and you don't hear children cry out "See the man with the gold teeth!" It is beautiful, durable and makes no display. I went with Marshall Webb to Stamford, to see the Wardwell Bros. fill a tooth for Webb. They introduced platinum gold to Webb in his own mouth! a lower molar on the left side. There were no more particular men in the world than the Wardwells; their work was all done to a nicety; they used the mechanical mallet; why don't they use the electric mallet? Certainly not because they don't understand it!

Marshall Webb himself was not always successful; Parmly Brown tells of many failures; they are but mortal men, and sometimes fail like the balance of us.

Dr. G. S. Staples, Sherman, Texas, said he had come from Texas to take a back seat, and listen and learn. There is a great difference in the manipulation of hard or soft gold. He was satisfied that twenty-fiour out of twentyfive operators ought to use soft foil, because it is much more difficult to use cohesive gold; a man who lacks thoroughness has no business to use it. The dentist is born, not made, and you can't make a dentist out of every young man. It will not do for a man who is nervous and impatient to undertake to fill teeth with cohesive gold. It requires more nerve than anything else. To attain success work must be thorough and don't stop till it is finished. The great cause of failure is lack of thoroughness. If you only expect to get twenty-five cents for it, do it perfectly anyhow. He said he was a hard foil man himself; had not bought five oz. of soft foil in twenty years.

Dr. Marshall, Little Rock, uses both foils. The trouble is due to the operator; to the power behind the plugger; not to the gold.

Dr. Freeman, Nashville, said the dark line at the margin was not always due to "the other end of the plugger." It was unprofitable to take up so much time in discussing the merits of cohesive and non-cohesive gold; the object is to preserve the teeth, and to do this we must use the material that we can use to the best advantage. If the gold slips, heat it a little and it becomes cohesive. In regard to the discoloration at the cervical margin, as long ago as in 1869 he read an article from Dr. H. S. Chase, of St. Louis, about the compatibility of tin to dentos, and began using it. On one occasion where he had put tin foil over a nearly exposed pulp, the tin slipped, and when finishing up the filling he noticed a dark crescent line at the margin, where a line of tin was visible, but that was certainly not an indication of decay! But he noticed later on that though those teeth had a predisposition to soften at the margins, the tooth in which the accident had occurred and the tin had slipped forward to the margin, was perfectly hard and sound; it appeared that tin was more adapted to cervical margins for its therapeutic effects. He was empirical to the extent of trying what was recommended by good authority, and continuing it when he found good results.

Tin does answer well at the margins, and for sensitive dentine; it also hardens the tooth structure of young teeth. Teeth that fail by other methods may be saved by tin linings, and the dark line at the margin, from the layer of tin does not indicate decay. If you put your gold where you want it, it will stay there if you don't disturb it. If you merely chunk in billets, and chunk them in the wrong place, you don't want them to stay. As Dr. Morgan said on another occasion it would be better if nine-tenths of our men would never attempt anything else but amalgam.

Dr. Winkler said that the great objection to tin was the discoloration it produces.

Dr. Freeman admitted the discoloration, but said that he always told his patients that it would discolor the tooth, but that it would preserve the tooth from future decay. A dark line at the margin, in his work, does not mean decay. The combination of gold and tin hardens the tooth, discoloring it by filling the tubuli with coloring matter. He knew that this was so, from practical tests and observation, but he wants to know why; he says, "I want the reason for the faith that is in me."

Dr. Louis Chisholm, Nashville, said that though a retired practitioner, no longer in the active ranks, he had listened with the greatest interest to the discussion, which called to mind the old fights in days gone by. He wished to call the attention of younger members to what had been as his *polar star* through life—that *demonstration* is the only witness that gives evidence to any fact of truth or science. When he was a young man, Dr. Shadoan had come forward with very hard foil. He made many fillings, which to the eye seemed perfect, grand; but in less than two years the cervical margins were permeated with rat holes; you could run your penknife under them; little sinks of moisture undermined it all. It requires such skill as not one man in a hundred possesses, to fill to the cervical wall perfectly. It is better to begin with soft gold fillings. If you have got the skill to use the other, all right; you may be the one in five hundred. But when retaining points are made in solid bed rock, and the gold built right at the margin, from one side to the other; moisture cannot enter, no matter if you leave a vacuum in the middle. Demonstration is the witness which will always testify to the truth.

Dr. Crawford said that though he did not ignore the advantages of non-cohesive or soft foil in certain cases, if he were compelled to confine himself to only one kind exclusively, he would take cohesive gold. Many fillings could be done with the latter that could not be done with the former. He said:—In what does the difference lie? Not in the make-up of the tooth; not in the shape of the cavity; not in the environment; it consists in the location and accessibility. If the cavity can be approached properly it can be filled with cohesive or with non-cohesive gold, as you choose. But where do you substitute amalgam for gold? It is where the cavity is so inaccessible that you cannot bring to bear the proper impact. This is the main point—the impact. The difficulty does not lie in the character of the material, nor in its introduction, but in the character of the impact. You want the minimun quantity of force. The means for complete condensation rest in the hand and arm which wield the instrument, far more than in any mechanical appliance and I predict that this will be recognized in less than a quarter of a century. If put to the test, it will be found that the proper use of the fingers, without mechanical aid, lessens labor, expedites operations, shortens the length of time necessary. It has been said that a greater weight of cohesive gold can be impacted than of non-cohesive; but gold is gold, and weighs the same the world over. If the cavity is absolutely full, the weight must be the same. The cardinal thought in the paper was the advantage of an established system, elevating the standard by which operating is controlled. In many large cavities the same result may be obtained—saving the tooth by either foil or with amalgam. By too severe malleting an inflammation of the gums is sometimes caused, bringing out products that are damaging to the tooth. The physical functions of the soft structures are interfered with, and the tooth becomes very sensitive. Many teeth are lost by being beaten to death with the mallet.

Dr. Crenshaw said that he was surprised to hear Dr. McKellops say that he preferred the mechanical mallet, because the blow of the electric mallet was too harsh. It was strange that he had not found out that the blow of the latter could be adjusted very exactly to any degree of force, making it as light or as heavy as you want. As to Bonwill's mechanical mallet, Dr. Bonwill had himself undertaken to demonstrate in a clinic, the practical perfection of his mallet, but he created such inflammation and trouble in the tooth that it had to be extracted. It may have stirred up an old slumbering abscess, but he had the opportunity of selecting his tooth. Having personally inspected the filling, he could say that it was not well condensed; it was very porous. Dr. Crenshaw asked Dr. Rollo Knapp to endorse this statement. The electric mallet can be adjusted to do what never has been done before. There will be no discoloration if the work is properly done with the electric mallet.

On motion the subject of operative dentistry was passed.

Dr. Salomon said that others had probably, like himself, been annoyed in the use of the emery strips, etc., in use for polishing fillings. He found that a ribbon of watch spring, heated in an alcohol lamp and drawn through shellac, and then dipped in rotton-stone, or silex, or corundum, would be found an excellent substitute; very elastic, and not liable to catch on the margin of the cavity and tear.

Dr. Catching related a remarkable case of dental development, the subject being Miss Julia Wells, of Atlanta, Ga. She was born August 6th, 1871, (a premature birth, of six months) of parents very sound, with no hereditary taints whatever. She was very delicate and very small. At the age of six months she began teething, and at seven months had a full set of small teeth. They were all lost within three months, and at eleven months she began cutting teeth again, at the age of fifteen months having a second set of teeth, which crumbled away like chalk. When two and a half years of age she weighed ten pounds and had a third set of teeth, which were a great source of irritation, causing inflammation and swelling of the face and under the lower jaw.

The mother carried the child to her dentist, Dr. T. T. Moore, of Columbus, S. C., (she was living there at that time,) to have the teeth removed. Dr. Moore thought best not to do so at that time; but she, persisting in her belief that they ought to come out, called on Dr. Boozer, who advised as Dr. Moore did. Not to be outdone, and to relieve her child, at her own risk, she borrowed forceps from Dr. Boozer and extracted twelve teeth of this set before releasing the child from her hold.

The teeth of the third set were all removed before she was four years old, after which her health improved rapidly. At seven years old she weighed thirty pounds. For several years she had no teeth, and then 3 or 4, shell like in form, appeared in front of the upper ridge, which were removed by the mother with her finger nails. Then she was toothless again until she was eleven years old, when she began teething again, cutting the fourth set, which are in her mouth sound and firm to-day. She is minus the two superior central incisors, one of which I removed on account of its position and one left superior bicuspid.

The lower jaw is minus the two right bicuspids, one left bicuspid and cuspid. The teeth were firmly fixed, apparently of good texture, not perfectly formed crowns. She uses them to good effect, although the jaws do not come together by about half an inch, and the lower jaw protrudes about one half inch. Up to two years of age she fed only from her mother's breast. Up to eight years of age she was given three times a day, cod liver oil and lime water.

Dr. Catching exhibited models of her mouth taken at different times.

Dr. Richards moved that copies of the casts and the history of this remarkable case be deposited with the Tennessee Historical Society, in memory of the present meeting.

Dr. Catching was requested to furnish the same.

Dr. Chisholm suggested that the President appoint a committee to consider the ways and means of establishing a permanent museum for the Southern Dental Association, in which might be preserved these, and other valuable specimens brought to the meetings of the Association.

Dr. Morgan said that this would necessitate a permanent location, and would tend to give the Society a local habitation as well as a name. The museum could be located at some central point where the Association could meet at regular intervals.

Drs. E. S. Chisholm, W. H. Richards, and G. V. S. Wright were appointed by the President as Committee on Museum.

A vote of thanks was tendered the Executive Committee, for the admirable manner in which they had performed their duties, and also to Dr. H. W. Morgan for his valuable assistance.

Dr. Freeman moved that the gavel be presented to the President, in testimony of the admirable manner with which he had wielded it.

Carried, with an amendment by Dr. Richards, that it be suitably engraved.

The President accepted the gift with brief but eloquent words of appreciation.

The selection of time and place of next meeting being now in order,—

Dr. McKellops said that if men from the West were expected to attend, a place must be selected where there would not be so much hot weather. They did not want to leave a hot place to come to one that was hotter. He hoped that point would be borne in mind. If they came South, they would prefer to come in the winter, when warm weather would be acceptable.

Dr. W. W. H. Thackston said that, while very grateful for the delightful social and professional privileges he had enjoyed at the present meeting, his special business was to present the claims of old Virginia. He had come as a delegate charged with that duty. If he might be allowed to make a suggestion, he would ask that Hygeia Hotel, Old Point Comfort, be selected as the place of meeting. It was not necessary to name all its attractions, but he would only refer to its delightful surf-bathing, and all the luxuries which earth, air and water combined can offer. He commended the Association to accept the love and hospitality of Old Virginia; that his people would do all they could to make them happy, if they would accept our hospitality and that of Hygeia Hotel.

Dr. Teague said that when such a man as Dr. Thackston could leave his lovely home, with all its comforts and luxuries, to come to the hottest place on earth to invite us to a paradise, how can we reject his invitation?

Dr. W. H. Morgan said that in justice to Nashville, he would say that when the Association was invited to Nashville it had been expected they would come in May, and every arrangement had been made to have lovely weather. He had had no voice in the change of time, and had not been able to control the weather.

On motion, the rules were suspended, and the Secretary instructed to cast the vote of the Association for Old Point Comfort, Va., as the next place of meeting.

Dr. Thackston expressed his extreme gratification at the success of his mission.

Dr. J. Hall Moore stated that the hotel was open the year round. In selecting the time of the meeting he would advise them not to go there in the month of June, as the hotel was usually very crowded then. He hoped that the time for meeting would be selected with reference to that of the International Congress on the 1st of September, and also so as not to conflict with the meeting of any other body, as had been unfortunately the case this year, conflicting with the National Dental Association.

Dr. McKellops thought the meeting should either follow close after, or precede the American Dental Association, also allowing time to go home before the Congress.

Dr. Catching said he had conferred with the chairman of the Dental Section of the Congress, and he thought it would be better to meet immediately before the Congress.

Dr. Teague moved that the selection of time, (and if found absolutely necessary a change of place also) be left in the hands ot the Executive Committee. Carried.

Dr. McKellops said that he had been in attendance at the meeting of the Congress in London. Americans had received a perfect ovation. If we went to Washington at the time of the coming Congress it would be necessary to go with pockets full of money.

Dr. Richards said that in case the hotel at Old Point Comfort could not be obtained, he would suggest Virginia Beach, as in every way delightful, with no undertow.

Dr. Thackston stated that in the invitation to Virginia the Association was uninstructed in choice of place. Fortress Monroe, Norfolk, Virginia Beach, Ocean View were all most enjoyable places in summer time.

The election of President being next in order, Dr. McKellops nominated Dr. B. H. Catching.

Dr. Beech nominated Dr. J. Hall Moore.

Dr. Crawford nominated Dr. J. Rolla Knapp.

Dr. Prewitt nominated Dr. W. W. H. Thackston.

Dr. Thackston said that he appreciated the sentiment which prompted his nomination, which made this the proudest moment of his life, he felt that he had had his day; that the measure of his claims upon the Association had been more than filled. That though it would be most gratifying to find himself again among such friends another year, he felt there were younger men better fitted to occupy the highest place in their gift. That he was an old man, on the down grade of life, and did not wish to stand as an obstacle in the path of younger men, who were more worthy than himself. He requested Dr. Prewitt to gratify him by making some other nominations.

Dr. Prewitt said that he could not agree to withdraw his nomination; that it was not true that he was passing into the sere and yellow leaf, for he was still active and efficient. He hoped that the Association would honor itself by voting for Dr. Thackston for President.

The nominations were declared closed, and Drs. Marshall and Johnson declared tellers.

On the first ballot Dr. Catching had fourteen votes; Dr. Thackston fourteen; Dr. J. Rollo Knapp five; Dr. J. Hall Moore eleven; blank one.

On the second ballot the name of Dr. Rollo Knapp was dropped. Dr. Thackston had nineteen votes; Dr. Catching eighteen, Dr. J. Hall Moore eight; blank one.

On the third ballot Dr. Thackston had twenty-three votes; Dr. Catching twenty; Dr. J. Hall Moore one; blank one.

Dr. Thackston having the majority of all the votes cast, on motion of Dr. Catching, the election was declared unanimous, and Dr. W. W. H. Thackston, Farmville, Virginia, announced as the next President of the Southern Dental Association.

Dr. Salomon nominated Dr. Catching for First Vice President, and on motion, the Secretary was instructed to cast the ballot.

Dr. J. R. Knapp was elected second Vice President.

Dr. W. H. Richards, third Vice President.

Dr. J. Y. Crawford, Corresponding Secretary.

Dr. Holliday, Recording Secretary, positively declining a re-election, Dr. L. P. Dotterer was elected to fill his place.

Dr. H. A. Lowrance was re-elected Treasurer.

Drs. J. Hall Moore, Richmond, Va.; E. S. Chisholm, Tuscaloosa, Ala.; J. R. Woodley, Norfolk Va.; Executive Committee.

On motion, adjourned to 2 P. M.

---

FOURTH DAY—SECOND SESSION.

Called to order at 2 P. M. Reading of the minutes dispensed with.

Dr. J. Hall Moore stated that he held a letter from Dr. R. Finley Hunt, addressed to the President of the Southern Dental Association, to appoint a committee of three to confer with a similar committee from the National Dental Association, the latter committee consisting of Drs. R. Finley Hunt, J. H. Coyle, E. S. Chisholm.

Dr. Teague said that he did not see that the Southern Association was called upon to recognize the National any more than the American Association. Let each one take care of itself.

Dr. McKellops did not see that the Southern Association had anything to gain from any connection with any other body. He had upheld the Southern through its darkest days; had helped to carry through our meeting when there was not a quorum in attendance. They were now prosperous and independent, and he thought it was better to remain so.

Dr. Salomon was opposed to appointing the committee. He would have each body stand on its own feet.

Dr. Catching said the object of the committee was apparently to prevent any further clashing of dates, but that was not likely to ever occur again.

Dr. Morgan said he did not want anything tending to any consolidation. It had once been proposed to wipe out both the American and the Southern Associations for the formation of a single National association, but no good had come of it. The Southern Association was now in good running order, ready to do efficient, harmonious work. He himself had been one of the hardest workers in its organization, though it was true he had at one time become discouraged, and had dropped out, but others had kept on, and now he was ready to put his shoulder to the wheel again. This was the representative Association of the entire Southern States. He said: There was never such a representative gathering of our brethren as we have here to day. I love my profession and I love my brethren. I have given it the ardor of my young manhood, and the vigor of my maturity, and I glory in its success.

Dr. Chisholm said he would give honor to whom honor was due. The Southern Association had been very small at one time. Then Dr. Morgan said: "You Southern people will not come to us; we will not come to you. We will build up the American Association." Now Dr. Morgan is ready is say, "*See how we apples swim*," but I am glad to know he is coming back to us, though he did once give us the cold shoulder. After the meeting at Augusta, Ga., Dr. Morgan said the papers read before the Southern Association were not worth the paper they were written on. I am glad he thinks better of us now. I was present at a meeting of the National Association four years ago, when on my way to preside over the Southern, but I have not attended a meeting since. I hope to live to see the Southern the leading representative body of the profession, and hope I shall live to rejoice in its fullest success.

After some further discussion, the vote was unanimous not to appoint a committee of conference.

The President, in brief but well chosen words, offered the Association his congratulations on the success of the meeting, thanking those who had so faithfully worked with him, supporting and sustaining him in his efforts. He then retired from the chair he had so ably filled, and the officers-elect were installed. The Association then adjourned to meet on Monday, at the office of Dr. W. H. Morgan, for clinics and final adjournment to Old Point Comfort, for the meeting of 1887.—*Southern Dental Journal.*

